# How I do it: Simpson grade I resection in a medial and inner ridge sphenoid wing meningioma

**DOI:** 10.1007/s00701-025-06626-0

**Published:** 2025-07-30

**Authors:** Yasmin Sadigh, Eva Joëlle Haasdijk, Victor Volovici

**Affiliations:** 1 Department of Neurosurgery, Erasmus MC Stroke Center, Erasmus MC University Medical Center, Dr Molewaterplein 40, 3015GD Rotterdam, The Netherlands; 2 Center for Complex Microvascular Surgery, Erasmus MC University Medical Center, Dr Molewaterplein 40, 3015GD Rotterdam, The Netherlands

**Keywords:** Anterior clinoid process, Skull base surgery, Sphenoid wing meningioma, Extradural clinoidectomy, Dural peeling

## Abstract

**Background:**

Extradural anterior clinoidectomy (EAC) and dural peeling of the lateral wall of the cavernous sinus (CS) are challenging skull base techniques that enhance exposure to anterior and middle cranial fossa lesions. Intimate knowledge of dural anatomy enables safe dissection and identification of critical neurovascular structures without cranial nerve deficit postoperatively.

**Methods:**

In a patient with a middle/inner ridge sphenoid wing meningioma, EAC and targeted dural peeling allowed for a Simpson grade I resection.

**Conclusion:**

EAC and targeted dural peeling enable a feasible Simpson grade I resection of a middle/inner ridge sphenoid wing meningioma, while minimizing neurovascular injury.

**Supplementary Information:**

The online version contains supplementary material available at 10.1007/s00701-025-06626-0.

## Relevant surgical anatomy

**Fig. 1:**
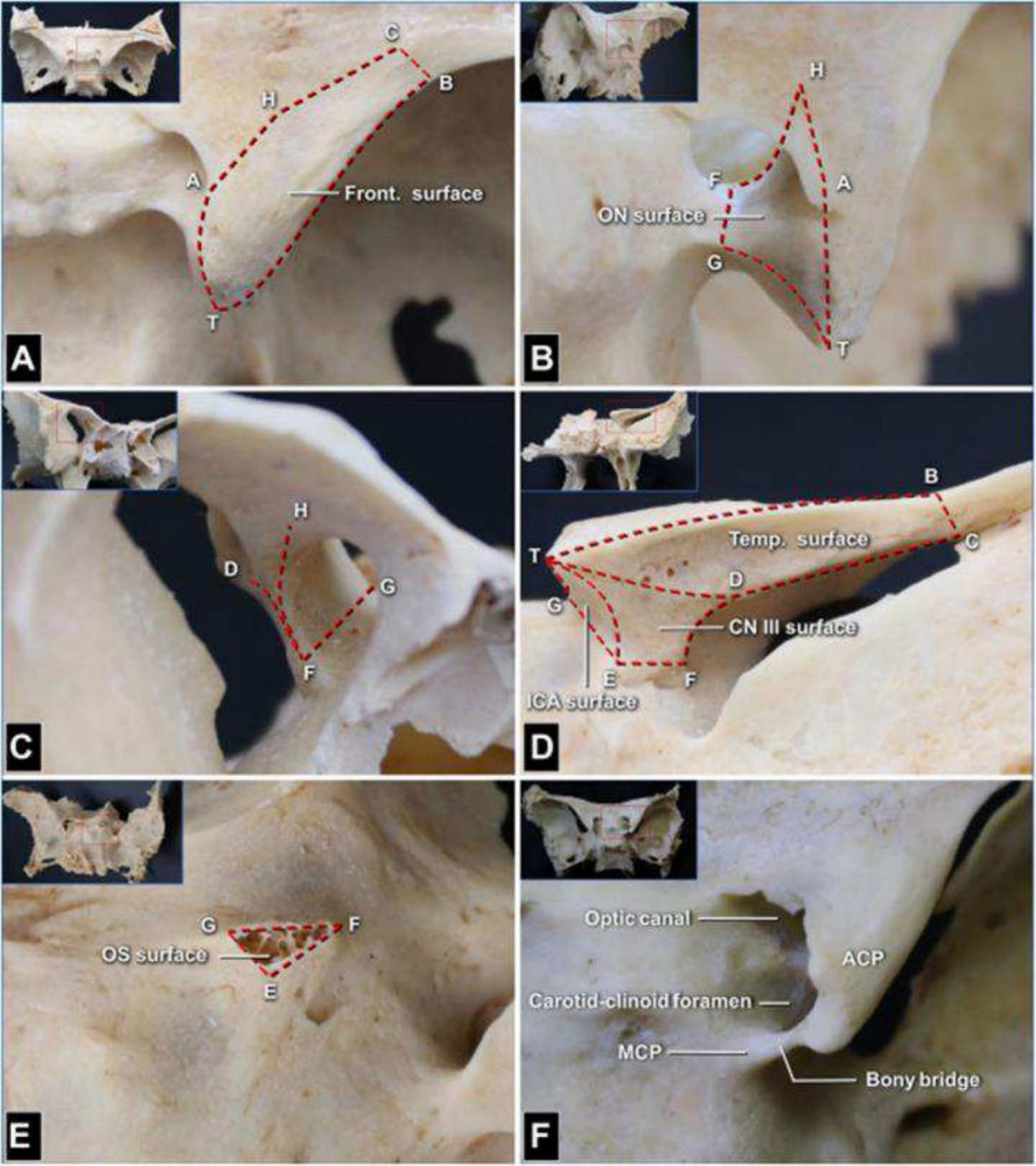
Dry skull specimens showing 9 key anatomic landmarks and the 6 surfaces on the anterior clinoid process (ACP; right side). The 9 anatomical landmarks are as follows: T, ACP tip; A, posterolateral point of optic canal roof on upper surface of lesser sphenoid wing (LSW); C, lateral endpoint of the superior orbital fissure (SOF; because the LSW is thin at this point, we use point C to mark both this point and its projection on the LSW surface); B, frontotemporal craniotomy performed to remove the frontal (Front.) and temporal (Temp.) flaps (point B is at the lateral end of the sphenoid ridge at the same depth of point C in the surgical corridor); H, anterolateral point of the optic canal roof on the upper surface of the LSW; D, transition point of neural and meningeal portions on superior border of SOF; E, posterolateral point of optic strut (OS) root on sphenoid body; F, anterolateral point of OS root on sphenoid body; G, superomedial point of OS root on sphenoid body. **A** Frontal surface (delineated by points T, A, H, C, and B). From the superior view, it is located on the upper surface of the LSW and between the upper edge of the lateral optic canal wall and sphenoid ridge. **B** and **C** Optic nerve (ON) surface. From the left posterior (**B**) and right anterior (**C**) views, the anterior part (delineated by points H, A, G, and F) of the ON surface is the lateral wall of the optic canal, and the posterior part (delineated by points A, T, and G) is the bony area facing the supralateral edge of the upper dural ring. **D** Temporal surface (delineated by points T, B, C, and D). From the posterior and lateral views, the temporal surface is located on the lateral aspect of the LSW, between the sphenoid ridge and upper edge of the meningeal portion of the SOF; cranial nerve (CN) III surface (delineated by points T, D, F, and E). This surface forms as the temporal surface extends inward over line TD. Its anterior edge (line DF) is the medial edge of the SOF; internal carotid artery (ICA) surface (delineated by points T, G, and E). A triangle is formed by the posterior edge of the OS root and the ACP tip. It expands inward into the ON surface and outward into the CN III surface. **E** OS surface (delineated by points G, E, and F). From the posterior and superolateral views, the OS surface is visualized as the triangular section of the surface of the OS root on the sphenoid body after removal of the ACP. **F** From the superior view, a carotid–clinoid foramen in another specimen is identified in the bony bridge between the ACP and the middle clinoid process. Reproduced with permission from Springer Nature Link (October 24, 2024) from Wu Y, Wu X, Zhang YZ, Wu YX, Zhu G, Li ZH, Luo JN, Xue YF, Cheng HB, Lv ZQ, Gao GD, Qu Y, Zhao TZ. A Six-Surface System to Describe Anatomy of Anterior Clinoid Process and Its Application in Anterior Clinoidectomy and Resection of Paraclinoid Meningioma. World Neurosurg. 2023 Oct;178:e777-e790

**Fig. 2:**
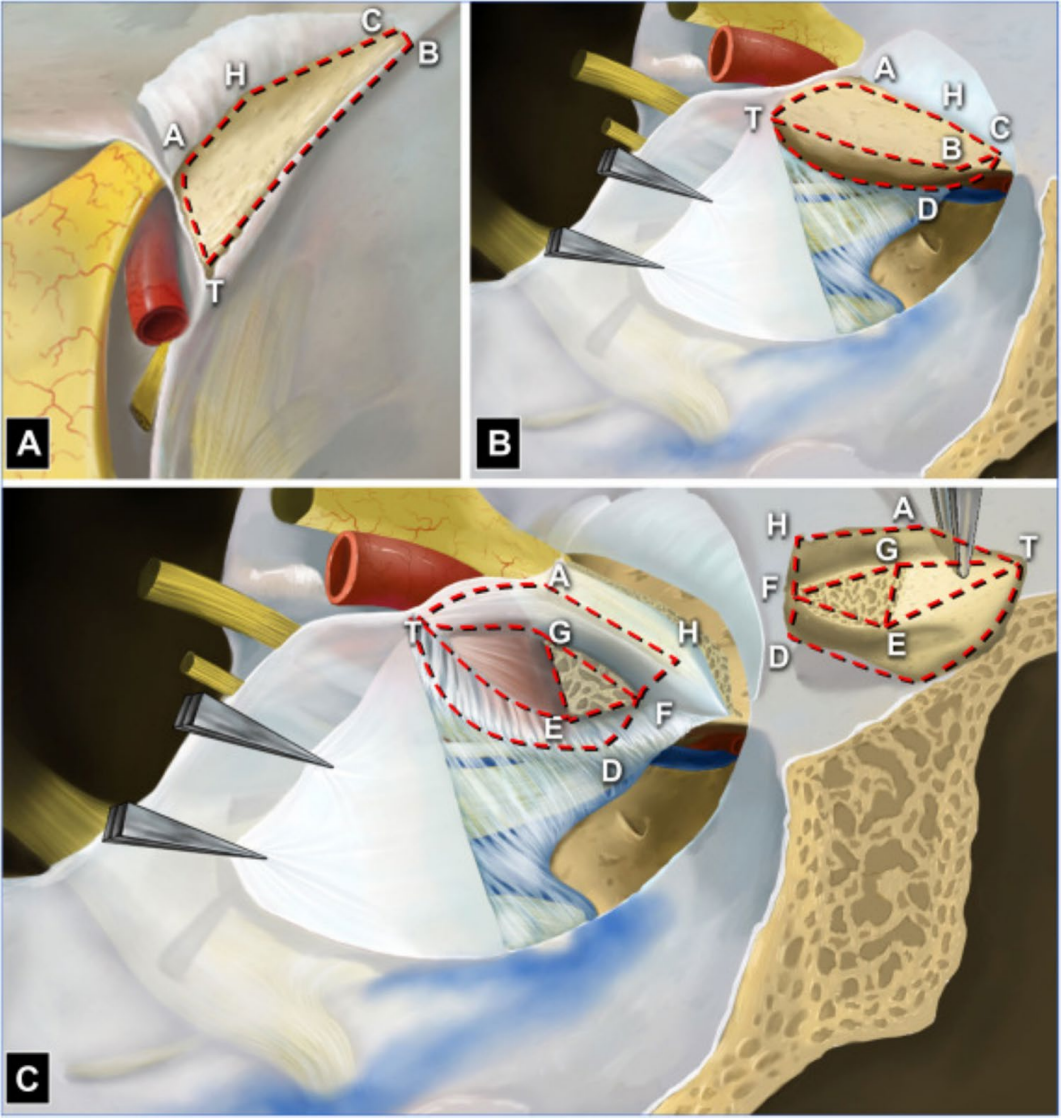
Schematic drawing of 6 surfaces and adjacent structures of the anterior clinoid process (ACP; right side). **A** From the superior view, the right ACP and adjacent structures are observed. The frontal surface (delineated by points T, A, H, C, and B) is exposed by uncovering the subfrontal dura from the upper surface of the lesser sphenoid wing (LSW). **B** From the lateral view, the meningo-orbital band (MOB) is cut, and the meningeal layer of the dura of the lateral wall of the cavernous sinus (LWCS) is peeled off to expose the temporal surface (delineated by points T, B, C, and D) of the ACP and the neural structures coursing in the LWCS to the SOF. **C** The ACP is turned up in a page-turning fashion to expose the dura corresponding to the ON (delineated by points H, A, T, G, and F), ICA (delineated by points T, G, and E), and CN III (delineated by points T, D, F, and E) surfaces, subdural structures, and the OS root. Reproduced with permission from Springer Nature Link (October 24, 2024) from Wu Y, Wu X, Zhang YZ, Wu YX, Zhu G, Li ZH, Luo JN, Xue YF, Cheng HB, Lv ZQ, Gao GD, Qu Y, Zhao TZ. A Six-Surface System to Describe Anatomy of Anterior Clinoid Process and Its Application in Anterior Clinoidectomy and Resection of Paraclinoid Meningioma. World Neurosurg. 2023 Oct;178:e777-e790

**Fig. 3:**
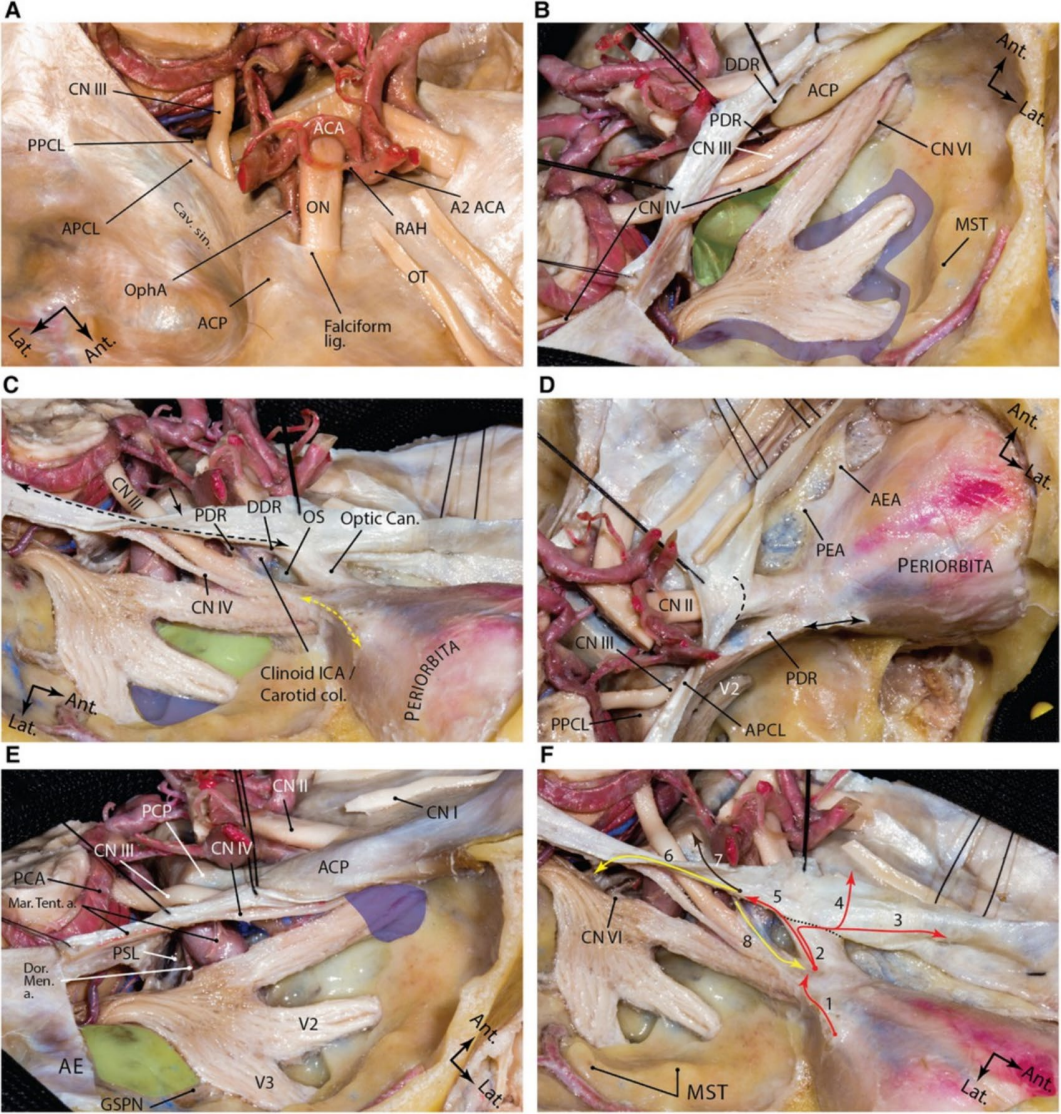
Dural anatomy of the middle fossa. **A** exposure of the middle fossa dura and its relationship with the dura and anatomic structures of the central skull base. **B** The meningeal and endosteal layers of the dural covering of the middle fossa are removed to expose the relevant neural relationships. Lifting the dura over the anterior clinoid process and along the lateral wall of the cavernous sinus. The proximal and distal dural rings are dural layers surrounding encompassing the anterior clinoid process. One may appreciate how trochlear nerve pierces the medial tentorial edge and enters the roof of the cavernous sinus. Meckel's cave orifice which is composed of meningeal layer of dura is widened around the trigeminal nerve root. This layer continues anteriorly as the roof of the cavernous sinus under the anterior clinoid process as the proximal dural ring. The green shaded area shows the Parkinson's triangle (the largest triangle of the cavernous sinus) through which the posterior genu of the cavernous carotid is exposed. The blue shaded area marks the laterotrigeminal venous system region. **C** Following anterior clinoidectomy, the relationship between the proximal and distal dural rings is better revealed. These layers converge at the root of the anterior clinoid process and are opened and continue laterally as the meningo-orbital band (dashed yellow double arrow) and anteriorly as the periorbita. Note the reflection of the meningeal layer of the dura (black arrow) along the tentorial edge (dashed black double arrow). Green and blue areas show and anteromedial and anterolateral triangles, respectively. **D** Upper view of the right cavernous sinus exposure and dural covering of the orbit along the cribriform plate. Black double arrow shows the amputated meningo-orbital band. Dashed line shows the location of the falciform ligament. **E** Exposure of the posterior aspect of the cavernous sinus and dural reflections around Meckel's cave entrance. Blue area shows the superior orbital fissure and green areas shows the middle fossa rhomboid (Kawase's). **F** Dural reflections in the paraclinoid region. 1, meningo-orbital band; 2, tip of the anterior clinoid process; 3, anterior fossa along the cribriform plate; 4, anterior fossa along the planum sphenoidale; 5, distal dural ring; 6, medial tentorial edge, anterior petroclinoid and petrosphenoid ligaments; 7, interclinoid ligament; 8, proximal dural ring. Black dashed line represents the location of falciform ligament. ACA, anterior cerebral artery; ACP, anterior clinoid process; ant., anterior; APCL, anterior petroclinoid ligament; cav., cavernous; CN, cranial nerve; lat., lateral; lig., ligament; ON, optic nerve; OphA, ophthalmic artery; OT, olfactory tract; PPCL, posterior petroclinoid ligament; RAH, recurrent artery of Heubner; sin., sinus. *(Courtesy of Barrow Neurological Institute. Used with permission.)*. Reproduced with permission (License CC BY 4.0) from Tayebi Meybodi A, Mignucci-Jiménez G, Lawton MT, Liu JK, Preul MC, Sun H (2023) Comprehensive microsurgical anatomy of the middle cranial fossa: Part I—Osseous and meningeal anatomy. Front Surg 10:1,132,774. https://doi.org/10.3389/fsurg.2023.1132774

The middle fossa (MF) is bordered anteriorly by the greater sphenoid wing (SW) and extending posteriorly behind the squamosal temporal bone along the petrous ridge. It houses essential neurovascular structures and communicates with the pterygopalatine and infratemporal fossae. The medial SW anchors the anterior clinoid process (ACP) [8]. The ACP is a bony prominence surrounded by critical structures, such as the optic nerve (ON), internal carotid artery (ICA), and ophthalmic artery (OphA) and is attached to the lesser SW by the roof of the optic canal (OC) and the optic strut (OS) (Fig. 1, 2) [9, 10]. Extradural anterior clinoidectomy (EAC) is a highly complex skull base technique, which provides safer and wider exposure for anterior and central skull base pathologies [2, 5–7]. Anatomical variations of the ACP range from an elongated ACP tip to the existence of a bony carotid ring, called the carotico-clinoid foramen, which forms when the ACP and middle clinoid processes are united by a bony bridge. The ACP may be pneumatized (communication of the ACP with the ethmoidal sinus) [1, 3, 4, 6]. The ACP is enveloped by dura and connected posteriorly to the petrous apex by the anterior petroclinoid fold (APF), a dural reflection forming part of the cavernous sinus (CS) roof and marks the beginning of the tentorial “root” [8]. This fold demarcates one border of the oculomotor triangle, through which the oculomotor nerve (nIII) enters the CS after piercing the dura lateral to the posterior clinoid process attached to the dorsum sellae [8]. Laterally, the greater SW and squamous temporal bone form the lateral floor of the MF [8]. Within the greater wing lie the foramen rotundum (FR), ovale (FO) and spinosum (FS). The FR allows passage of the maxillary nerve (nV2) to the pterygopalatine fossa [9]. FO transmits the mandibular nerve (nV3), the accessory meningeal artery, and the lesser petrosal nerve [9]. FS admits the middle meningeal artery (MMA) [9]. Figure 3.

## Description of the technique

**Fig. 4:**
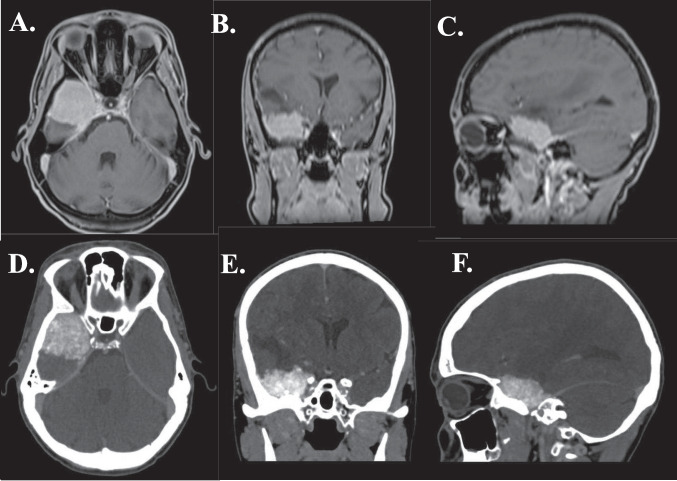
**ABC:** MRI scan (T1W sequence) of patient with medial sphenoid wing meningioma with optic nerve involvement. **DEF:** CT scan of patient with medial sphenoid wing meningioma, compressing the optic nerve

Video 1 shows a demonstration of EAC and targeted dural peeling in case with middle/inner ridge SW meningioma (Fig. 4).

**Fig. 5:**
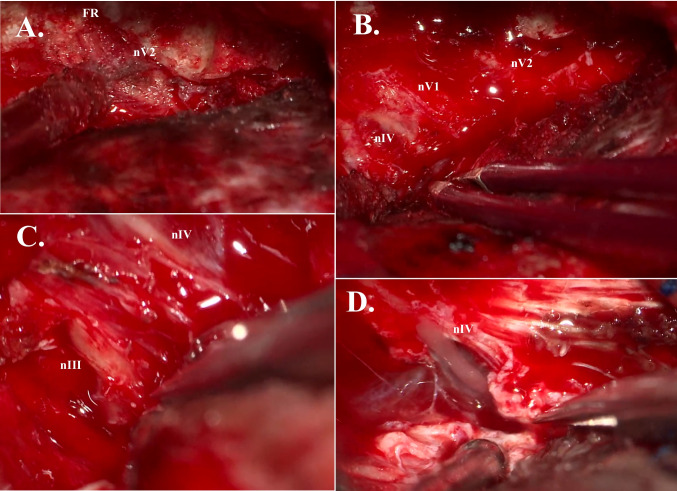
**A:** Extradural unroofing of foramen rotundum (FR), exposing maxillary nerve (nV2). **B:** Extradural view of trochlear nerve (nIV), ophthalmic nerve (nV1), and maxillary nerve (nV2). **C:** Intradural dissection of oculomotor nerve (nIII) and trochlear nerve (nIV) from dural and tumoral adhesions. All dural adhesions were removed. No oculomotor or trochlear palsy was observed postoperatively. **D:** Intradural view of fully skeletonized trochlear nerve (nIV)

The patient is placed in supine position, head rotated approximately 45 degrees. A curvilinear incision, starting at the upper edge of the zygomatic roof and extending just above the frontozygomatic suture, is made. After skin incision and interfascial dissection, the temporalis muscle is freed from its insertion and reflected inferiorly to ensure superior-anterior exposure. After craniotomy, the edge of the greater SW is drilled with a 4.0 mm coarse diamond drill and the periorbita is exposed. The meningo-orbital band is skeletonized, coagulated, and cut. By having the periorbita exposed at the level the CS-superior orbital fissure junction, the plane between the dura propria of the temporal lobe and CS lateral wall can be identified. Dissection continues until nV2 is freed at the border of the FR, over Meckel’s cave to the lateral border of nV3, just beyond the dural tail noticed in the free border of the tentorium. A combination of micro-scissors and a number 4 Penfield micro is used. Sharp dissection and intimate anatomical knowledge of the dural folds is important in areas where the dural layers are heavily intertwined, such as between the ophthalmic nerve (nV1) and nV2. After the dura is peeled posteriorly, the ACP is disconnected from the APF. The dura is peeled further posteriorly, and the FR is unroofed to expose nV2 (Fig. 5A). The MMA and middle meningeal vein are exposed, coagulated and cut.

**Fig. 6:**
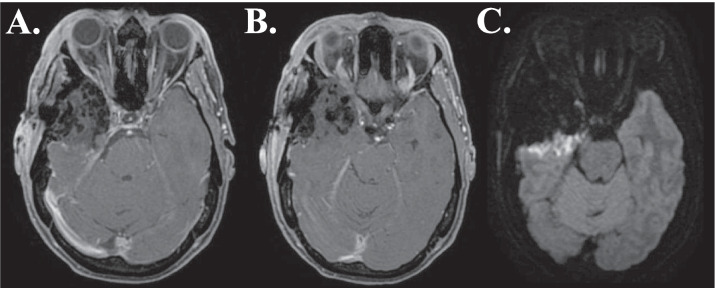
**AB:** Postoperative (< 48 h) MRI scan (T1W sequence) of patient with medial sphenoid wing meningioma, showing postoperative changes and Simpson grade I resection. **C:** Postoperative (< 48 h) MRI scan (DWI sequence) of patient with medial sphenoid wing meningioma, showing mild diffusion restriction at the pia-meningioma interface

The OC is identified subfrontally and intraorbitally. The roof of the OC is drilled using a 2.0 mm cutting burr and special Friedman micro-rongeurs. The ON (still protected in its dural sheet) is exposed and ICA is identified. The dura covering the ACP is dissected, allowing for internal decompression with the drill and rongeur. The ACP tip is removed by disconnecting it from the OS and dural adhesions. Intradural approach is initiated; the outer tumoral border is detached from the cortex and tumoral tissue is debulked. Space inferior to the tumor is widened and tumor manipulation is possible without traction. The plane of dissection between the temporal lobe and tumor, essential for preserving posterior M4 and P4 branches, is better visualized (Fig. 6).

The APF and the dura of the lateral wall of the CS covering nIII and the trochlear nerve (nIV) are sharply incised. Removal proceeds from anterior to posterior, tangential to the nerves (Fig. 5C-D). Hemostasis in the CS is achieved with fibrin glue, gelfoam soaked in tranexamix acid, and FloSeal.

## Indications

EAC is indicated to gain a wider exposure when treating anterior and central skull base pathologies, including OphA, ICA and basilar apex aneurysms, and tumors such as ACP, planum sphenoidale, middle/inner SW and parasellar meningiomas, and craniopharyngiomas. Dural peeling from the MF is essential in a variety of skull base approaches, easing access to deep neurovascular structures.

## Limitations

Factors limiting the possibility of achieving Simpson I are:

- Extension into the sphenoidal sinus along the clinoidal ICA implies invasion of the distal dural ring. While the ring itself can be dissected from the ICA, the fibers of the ring may be highly adherent.
- CS extension, especially in cases where tumor engulfs the cavernous ICA, as the dissection plane is difficult to define.
- Posterior CS extension entails complete removal of the petrous apex and Gruber’s ligament. Otherwise, lesions of the abducens nerve are highly likely.

## Avoiding complications

EAC can prevent mechanical injury to neurovascular structures as the dura remains intact. To avoid thermal injury to the ON, caused by heat of the drill and bipolar, timely irrigation is crucial. Tangential sharp dissection along nIII and nIV with as little manipulation as possible in the early dissection phase is essential. In cases with ACP pneumatization and Simpson I resections, harvested abdominal fat can be used to close the dural gap or communicating sinuses to prevent cerebrospinal fluid (CSF) leakage.

## Specific information for the patient

A comprehensive overview of risks and benefits of the surgical approach, based on the anatomy, co-morbidities, and neurosurgeon’s experience should be provided to the patient, prior to shared decision-making. Unfavorable anatomy needs to be discussed with the patient accompanied by additional intraoperative risks.

## Key points

1. The ACP is surrounded by the ON, ICA and OphA, which makes EAC challenging.
2. EAC enhances the surgical view.
3. OC unroofing and OS removal are required to detach the ACP from the inner sphenoid ridge.
4. Anatomical variations such as the pneumatized ACP and bony bridges can complicate surgery and increase risk of CSF leakage or vascular injury.
5. Dural peeling and resection of the dural folds is essential to achieve Simpson grade I resection.
6. Peeling the lateral wall of the CS helps expose foramina (FR, FO, FS) and cranial nerves (nV1, nV2).
7. Intradural steps include tumor debulking and careful detachment from surrounding structures, preserving all M4 and P4 branches.
8. Dura should be incised sharply, tangential, and parallel to the nerves from anterior to posterior.
9. The approach should be tailored to the amount of dural tail present, and a Simpson grade I should only be attempted when dural resection on the nerves is necessary without extensive manipulation.
10. CSF leakage due to sinus communication and absence of affected dura can be avoided by harvesting and placing abdominal fat grafts in the defect.

## Supplementary Information

Below is the link to the electronic supplementary material.

Supplementary file1 (MP4 298511 KB)

## Data Availability

No datasets were generated or analysed during the current study.

## Abbreviations

MF: Middle Fossa
SW: Sphenoid Wing
ACP: Anterior Clinoid Process
ON: Optic Nerve
ICA: Internal Carotid Artery
OphA: Ophthalmic Artery
OC: Optic Canal
OS: Optic Strut
EAC: Extradural Anterior Clinoidectomy
CS: Cavernous Sinus
APF: Anterior Petroclinoid Fold
nIII: Oculomotor Nerve
nIV: Trochlear Nerve
nV1: Ophthalmic Nerve
nV2: Maxillary Nerve
FR: Foramen Rotundum
FO: Foramen Ovale
FS: Foramen Spinosum
MMA: Middle Meningeal Artery
MRI: Magnetic Resonance Imaging
CSF: Cerebrospinal Fluid
